# Efficacy and safety of traditional Chinese medicine for the treatment of epilepsy by wind quenching and phlegm resolving: A systematic review and meta-analysis

**DOI:** 10.1097/MD.0000000000039942

**Published:** 2024-10-25

**Authors:** Yufen Cai, Xiaofang He, Liting Ao, Yibo Liu, Yanju Zhang, Hui Yang, Lin Zhang

**Affiliations:** a Guilin Hospital of Traditional Chinese Medicine, Guangxi, China; b Guizhou University of Chinese Medicine, Guiyang, China; c Department of Pediatric Intensive Care Unit, Guizhou Provincial People's Hospital, Guiyang, China; d Wuxi School of Medicine, Jiangnan University, Beijing, China; e Department of Neurology, The Second Affiliated Hospital of Guizhou University of Chinese Medicine, Guiyang, China.

**Keywords:** epilepsy, meta-analysis, systematic review, traditional Chinese medicine, wind-quenching and phlegm-resolving therapy

## Abstract

**Background:**

Using wind-quenching and phlegm-resolving (WQPR) therapy for epilepsy has yielded beneficial results in various clinical studies. However, a comprehensive analysis of the WQPR approach to epilepsy has not yet conducted to date. This study aimed to evaluate the effectiveness and safety of the WQPR method in traditional Chinese medicine (TCM) for epilepsy.

**Methods:**

Eight databases, including The Cochrane Library, Web of Science, PubMed, Embase, Chinese Biomedical Database, Chinese National Knowledge Infrastructure, Chinese Science and Technology Periodical Database (VIP), and WanFang Database, were comprehensively searched to include randomized controlled trials (RCTs) investigating the WQPR approach in epilepsy. Quality was evaluated by the Cochrane Handbook for Systematic Reviews of Interventions, and meta-analysis was conducted using the RevMan 5.4 and Stata 14.0 software. For the outcome indicators with the number of studies ≥ 10, funnel chart and Egger test were used to evaluate the bias, and the evidence quality was evaluated according to GRADEpro system.

**Results:**

We included 19 randomized controlled trials with 1475 participants. Compared to the control group, the WQPR approach showed clinical efficacy for epilepsy (odds ratio = 3.23, *95%* confidence interval [*CI*] [2.19, 4.77], *Z = *5.90, *P* < .00001), reduced seizure frequency (standardized mean differences = ‐1.24, *95% CI* [‐1.62, −0.85], *Z* = 6*.26*, *P* < .00001), shortened seizure duration (standardized mean differences = ‐2.07, *95% CI* [‐2.99, ‐1.14], *Z *= 4.39, *P* < .0001), improved patient’s quality of life (mean difference = 2.60, *95% CI* [2.16, 3.03], *Z = 11.62*, *P* < .00001), and ameliorated TCM syndromes (mean difference = ‐4.37, *95% CI* [‐6.19, ‐2.56], *Z* = 4.72, *P* < .00001). The reduced rate of adverse reactions (odds ratio = 0.56, *95% CI* [0.37, 0.85], *Z* = 2.71, *P* = .007).

**Conclusion:**

WQPR therapy appears to be an effective and safe approach for treating epilepsy, increasing clinical efficacy, reducing seizures’ frequency and duration, improving patients’ quality of life, ameliorating TCM syndromes, and reducing adverse reaction rates.

## 1. Introduction

Epilepsy, a chronic recurrent disease, is characterized by the transient manifestation of abnormal synchronous neuronal discharges, resulting in seizures.^[1]^ According to epidemiological data, the prevalence of epilepsy in the world is approximately 0.6% to 1.0%,^[2]^ about 50 million to 70 million,^[3,4]^ and China alone has >9 million epilepsy patients. Of them, 5 to 6 million experience active seizures, and 650,000 to 700,000 new cases are reported every year.^[5]^ The intricate pathogenesis of epilepsy involves an imbalance between the central nervous system’s excitatory and inhibitory processes, which is associated with ion channel abnormalities, neurotransmitter imbalances, oxidative stress, and inflammatory responses.^[6]^

Current therapeutic approaches for epilepsy primarily rely on antiepileptic drugs (AEDs).However, more than 20 AEDs have been developed based on different pathogenic mechanisms. However, approximately 30% of patients exhibit poor responses to rational and sufficient AED treatment, thereby leading to drug-resistant epilepsy, also known as refractory epilepsy.^[7]^ The recurrent and challenging nature of epileptic seizures not only places a significant economic burden on society and families, but also profoundly impacts the patient’s psychological, cognitive, and behavioral aspects.

The treatment of epilepsy using Traditional Chinese medicine (TCM) dates back a thousand years. TCM herbal formulations exhibit the synergistic activities of multiple components and exert a holistic effect through various targets and pathways. Furthermore, TCM offers a practical and cost-effective approach with high research potential and promising application prospects for various complex diseases. The research and development of AEDs derived from traditional Chinese medicine has become a new research direction in the treatment of refractory epilepsy in recent years.

In TCM, the origin of epilepsy is attributed to several internal and external factors like disruptions in organ functions and Yin-Yang imbalances. Furthermore, the resultant activities, that is, the ascent of Wind, Fire, Qi, and Phlegm, as well as blood stasis, obstruct the opening of the orifices, block meridians, and cause Qi circulation disorders, thereby leading to loss of control over the spirit and the onset of the disease. If the disease is left untreated for a long time, the internal organs become increasingly deficient and the epilepsy recurs, becoming a chronic disease.^[8]^ Wind-phlegm-obstructing orifices are the most common TCM pattern in epilepsy, with the treatment principle focusing on the wind-quenching and phlegm-resolving (WQPR) approach. Hence, this study aimed to systematically evaluate the efficacy and safety of TCM’s WQPR approach in the treatment of epilepsy according to the Cochrane Handbook and GRADEpro system.

## 2. Materials and methods

This meta-analysis adhered to the Preferred Reporting Items for Systematic Reviews and Meta-Analyses (PRISMA) guidelines. The study protocol was registered on the PROSPERO platform (registration number CRD 42023457958). However, ethical considerations were not applicable to this study.

### 2.1. Literature search

Two independent researchers (HXF and ALT) conducted a comprehensive search across 8 databases, including The Cochrane Library, Web of Science, PubMed, Embase, Chinese Biomedical Database, Chinese National Knowledge Infrastructure, VIP, and WanFang Database. The search aimed to collect clinical randomized controlled trials (RCTs) related to the use of the WQPR approach in treating epilepsy and was from their inception to July 2023. The search terms included keywords: “Epilepsy,” “Seizures,” “Epilepsy Syndrome,” “Wind,” “Phlegm,” “Wind-Quenching and Phlegm-Resolving Method,” and “Traditional Chinese Medicine.”

### 2.2. Inclusion and exclusion criteria

#### 2.2.1. Inclusion criteria

(1) All published RCTs investigated the WQPR approach in treating epilepsy, regardless of language (Chinese or English).
(2) Participants without limitations of age, sex, race, region, or epilepsy type who fulfilled both Western clinical and TCM diagnostic criteria for epilepsy and seizure disorders, respectively. Western diagnostic criteria followed the International League Against Epilepsy guidelines of 1981, 1989, 2001, and 2017, whereas TCM diagnostic criteria were based on documents from the Chinese Ministry of Health in 1993^[9]^ and the State Administration of TCM in 1994.^[10]^
(3) The experimental group received herbal medicine primarily based on the WQPR approach, without any restrictions on the drug composition, formulation, or dosage. The control group received conventional AEDs either alone or in combination.
(4) Observation indicators included at least one of the following parameters: clinical efficacy, seizure frequency, seizure duration, 31-item Quality of Life in Epilepsy Inventory (QOLIE-31) questionnaire, and adverse reactions.

#### 2.2.2. Exclusion criteria

(1) Experimental groups combined external treatments like acupuncture, moxibustion, or herbal massages, while the control groups used herbal formulations with TCM components with conventional AEDs.
(2) Exclusion of animal and cell experiments, case reports, experience and theory summaries, conference papers, and systematic reviews.
(3) Outcome indicators outside the scope of this study.
(4) Self-controlled RCTs.
(5) Studies with flawed experimental designs, inaccessible full texts, or incomplete data.

### 2.3. Literature screening

Two researchers (CYF and LYB) independently screened and cross-verified the literature. Any disagreements were resolved through discussion with a third researcher (YH). The screening process involved the following steps: utilizing the NoteExpress software to eliminate duplicate literature, reviewing titles and abstracts to exclude irrelevant studies, reading full texts to choose studies based on inclusion and exclusion criteria, and cross-checking the accumulated studies.

### 2.4. Data extraction

Two researchers (HXF and ZYJ) independently extracted the data by including information like first author, publication year, patient demographics, sample size, treatment interventions, TCM syndrome differentiation, TCM treatment principles, herbal medicine composition, treatment duration, and outcome indicators. Cross-checking was performed and if there were disagreements, the third researcher (ZL) was asked to discuss and resolve them together. Finally, the data were integrated into MS Excel.

### 2.5. Quality assessment of literature

Two researchers (CYF and HXF) independently used the 7-item Cochrane 5.1.0 Bias Risk Assessment Tool to assess the quality of included literature. This tool evaluates random sequence generation (selection bias), allocation concealment (selection bias), blinding of participants and personnel (performance bias), blinding of outcome assessment (detection bias), incomplete outcome data (attrition bias), selective reporting (reporting bias), and other potential sources of bias (e.g., small sample size, baseline imbalance). Each item was rated as “low risk,” “unclear,” or “high risk.” Cross-checking was performed and if there were disagreements, the third researcher (ZL) was asked to discuss and resolve them together.

### 2.6. Quality of evidence

The GRADEpro system was used to judge the quality of evidence for the outcome indicators. Five factors that may reduce the quality of evidence in the intervention system evaluation of GRADE system: Risk of bias, Inconsistency, Indirectness, Imprecision, and Other considerations. The levels of evidence quality were rated at 4 levels: high, medium, low, and very low. The level represents the strength of the evidence.

### 2.7. Statistical analysis

Review Manager 5.4 software was used for the statistical analysis. For continuous and categorical data, the standardized mean differences (SMD), mean difference (MD), and odds ratio (OR), were used as the effect size measures with 95% confidence intervals (CI), respectively. The Chi-square test was employed to analyze the data from the included studies with a significance level of α = 0.1. Heterogeneity was quantitatively assessed using the *I*^2^ test. Significant heterogeneity was assumed when *P* < .1 and *I*^2^ > 50%. Subgroup analysis was conducted to explore the causes of heterogeneity, whereas sensitivity analysis was conducted to assess the stability of the result. Conversely, a fixed-effects model was used for analysis if *P* > .1 and *I*^2^ ≤ 50%, denoting homogeneity or minor heterogeneity. Statistical significance was considered at *P* < .05 or *P* < .01. Publication bias was assessed when outcome indicator studies were ≥10. Funnel plots and Egger plots were drawn using RevMan 5.4 and Stata 14.0 software, and publication bias was analyzed according to the degree of symmetry of the funnel plots and the Egger test.

## 3. Results

### 3.1. Literature retrieval results

**Figure 1.**
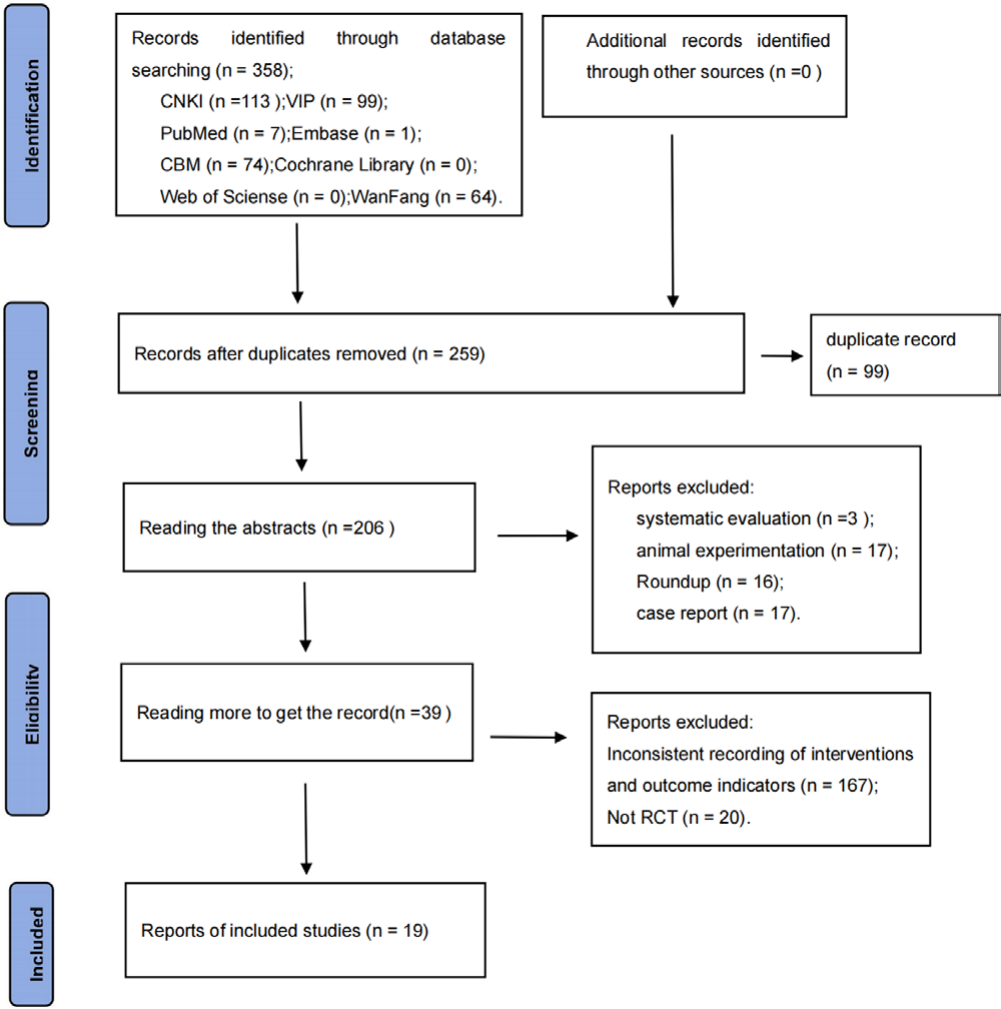
Flowchart depicting the study selection process.

A total of 358 articles were retrieved through electronic search. Furthermore, 99 duplicate articles were removed using the NoteExpress software. The remaining literature review excluded 251 irrelevant articles. Ultimately, 19 articles that met the inclusion criteria were included in the analysis (Fig. 1).

### 3.2. Basic characteristics of included studies

Nineteen selected studies encompassed 1475 participants, with 738 and 737 participants in the experimental and control groups, respectively. The clinical characteristics of the specific literature are shown in Table 1. The experimental group received a combination of TCM WQPR with conventional AEDs, while the control group received conventional AEDs either alone or in combination. Although the composition of the Chinese medicines used in the treatment group was not entirely consistent, the overall principle was to WQPR (Tables 2 and 3).

**Table 1:** Characteristics of the included RCTs.

First author	Years	Number of samples		Intervention measures		Course of treatment (Mo)	Outcome indexes
		Treatment group	Control group	Treatment group	Control group		
Chen Yan^[11]^	2019	32	32	Dingxianwan + C	Sodium valproate	6Mo	1, 3, 4
Li Huan^[12]^	2022	33	33	Dingxianwan + C	Oxcarbazepine	3Mo	1, 2, 3, 5, 6
Xiao^[13]^	2022	46	46	Dingxianwan + C	Conventional western medicine	3Mo	3, 4, 6
Yao^[14]^	2017	35	35	Dingxian Xifeng Decoction + C	Carbamazepine/Sodium valproate	3Mo	1, 2, 3
Wang Mei^[15]^	2017	30	30	Self-designed Phlegm-Resolving and Wind-Quenching Decoction + C	Carbamazepine/Sodium valproate	3Mo	2, 5
Zhou^[16]^	2015	30	30	Self-designed Phlegm-Resolving and Wind-Quenching Decoction + C	Conventional western medicine	6Mo	5
Feng^[17]^	2016	41	40	Self-designed Phlegm-Resolving and Wind-Quenching Decoction + C	Conventional western medicine	3Mo	5
Lu Lingdan^[18]^	2015.6	30	31	Self-designed Phlegm-Resolving and Wind-Quenching Decoction + C	Conventional western medicine	3Mo	5
Liu Xuexue^[19]^	2015	30	30	Jiawei Chaihu plus Longgu Muli particle + C	Sodium valproate	12Mo	1, 5, 6
Chen Ailing^[20]^	2019	39	41	Jiawei Chaihu Shugan Powder + C	Carbamazepine	2Mo	1, 2, 3, 4, 5, 6
Wang Congli^[21]^	2013	40	40	Zhenjing Zhixian Pill + C	Phenytoin sodium	6Mo	1, 3, 6
Cao^[22]^	2015	23	22	Self-designed Phlegm-Resolving and Wind-Quenching Decoction + C	Conventional western medicine	3Mo	2, 5, 6
Wu^[23]^	2015	30	30	Houshi Heisan + C	Sodium valproate	6Mo	1, 2, 5
Dai^[24]^	2017	45	45	Chaibei Zhixian decoction + C	Anti-ep conventional	6Mo	1, 3, 6
Hu^[25]^	2018	82	81	Dianxiankang Capsules + C	Oxcarbazepine	3Mo	1, 3, 4, 6
Wang Fu^[26]^	2021	37	37	Dianxiankang Capsules + C	Sodium valproate	2Mo	3, 4
Mao^[27]^	2018	46	46	Xianyu Capsules + C	Left ethyl latasi	6Mo	1, 3, 6
Ji^[28]^	2018	41	40	Xianyu Capsules + C	Sodium valproate	2Mo	1, 3, 6
Liu Haiying^[29]^	2020	48	48	Kangxian Jiejing decoction + C	Oxcarbazepine	6Mo	1, 3, 4, 6

1. total effective rate; 2. TCM symptom scores; 3. frequency of epileptic seizures; 4. duration of seizures; 5. QOLIE-31 score; 6. adverse reactions.

C = control group.

**Table 2:** Composition of included studies’ herbal formulations.

Inclusion of studies	Name of the formula	Main components of the formula (Chinese Pinyin name)
Chen Yan^[11]^	Dingxianwan + C	Chuanbeimu, jiangcan, chenpi, dannanxing, danshen, dengxincao, fuling, fushen, tianma, hupo, jiangbanxia, quanxie, shichangpu, shengjiang, maidong, shengshaishen, gancao, zhusha
Li Huan^[12]^	Dingxianwan + C	tianma, chuanbeimu, jiangbanxia, fuling, fushen, dannanxing, shichangpu, quanxie, jiangcan, hupo, chenpi, yuanzhi, danshen, maidong, zhusha
Xiao^[13]^	Dingxianwan + C	dannanxing, tianma, jiangcan, chenpi, danshen, dengxincao, jiangbanxia, fuling, hupo, fushen, quanxie, shichangpu, shengjiang, chuanbeimu, maidong, shenshaishen, gancao, zhusha
Yao^[14]^	Dingxian Xifeng Decoction + C	tianma, gouteng, dannanxing, chantui, banxia, chenpi, fuling, shichangpu, yuanzhi, quanxie, jiangcan, shenglonggu, shengmuli, yujin, maidong, beimu, baiziren, gancao
Wang Mei^[15]^	Self-designed Phlegm-Resolving and Wind-Quenching Decoction + C	dannanxing, fuling, zhebeimu, gouteng, jiangcan, shichangpu
Zhou^[16]^	Self-designed Phlegm-Resolving and Wind-Quenching Decoction + C	dannanxing, jiangcan, gouteng, yujin, shichanpu, zhebeimu, fuling, banxia
Feng^[17]^	Self-designed Phlegm-Resolving and Wind-Quenching Decoction + C	dannanxing, shichangpu, jiangcan, fuling, fabanxia, gouteng, zhebeimu
Lu Lingdan^[18]^	Self-designed Phlegm-Resolving and Wind-Quenching Decoction + C	dannanxing, fabanxia, jiangcan, gouteng, shichanpu, zhebeimu, fuling
Liu Xuexue^[19]^	Jiawei Chaihu plus Longgu Muli particle + C	chaihu, huangqin, dangshen, banxia, shengjiang, dazao, guizhi, fuling, longgu, muli, zhenzhumu, tianma, jiangcan, zhigancao
Chen Ailing^[20]^	Jiawei Chaihu Shugan Powder +C	chaihu, chuanxiong, xiangfu, shichangpu, dannanxing, chenpi, shaoyao, zhike, banxia, fuling, gancao
Wang Congli^[21]^	Zhenjing Zhixian Pill + C	quanxie, lingyangjiao, zheshi, jiangcan, zhenzhumu, gouteng, tianzhuhuang, dannanxing, shichangpu, danshen, yujin, baishao, lianzixin, qingdai, dahuang, bohe, zhishi, gancao
Cao^[22]^	Self-designed Phlegm-Resolving and Wind-Quenching Decoction + C	banxia, baizhu, tianma, gouteng, fuling, chenpi, shichangpu, yuanzhi, dannanxing, jiangcan, shijueming, zhigancao
Wu^[23]^	Houshi Heisan + C	juhua, baizhu, xixin, fuling, muli, jiegeng, fangfeng, renshen, fanshi, huangqin, danggui, ganjiang, chuanxiong, guizhi
Dai^[24]^	Chaibei Zhixian decoction + C	zhebeimu, chaihu, muli, shichangpu, tianma, dilong
Hu^[25]^	Dianxiankang Capsules + C	tianma, shichangpu, jiangcan, dannanxing, chuanbeimu, danshen, yuanzhi, quanxie, maidong, danzhuye, shengjiang, hupo, renshen, bingpian, rengongniuhuang
Wang Fu^[26]^	Dianxiankang Capsules + C	shichangpu, tianma, jiangcan
Mao^[27]^	Xianyu Capsules + C	huangqi, yuanzhi, danshen, dangshen, tianma, chaihu, danggui, gouteng, suanzaoren, yujin, dannanxing, shichangpu, baifuzi, liushenqu, jiangcan, gancao
Ji ^[28]^	Xianyu Capsules + C	huangqi, dangshen, danshen, chaihu, suanzaoren, yuanzhi, tianma, shichangpu
Liu Haiying^[29]^	Kangxian Jiejing decoction + C	shengcishi, niuxi, fuling, dannanxing, dilong, banxia, jiangcan, jianghuang, shengdahuang, juhong, shichangpu, chuanxiong, guizhi, shenshaishen, gancao, chantui, chenxiang

**Table 3:** Chinese herbs and efficacy based on frequency of usage in the 19 study prescriptions.

English name	Latin name	Chinese Pinyin name	Frequency of usage
Phlegm-resolving herbs			
Bile Arisaema	Arisaema Cum Bile	Dannanxing	14
Pinellia Tuber	Pinelliae Rhizoma	Banxia	11
Fritillary bulb	Fritillariae Bulbus	Beimu	10
Tangerine Peel	Citri Reticulatae Pericarpium	Chenpi	6
Wind-quenching herbs			
Tall Gaxtraodia Tuber	Gastrodiae Rhizoma	Tianma	11
Stiff Silkworm	Bombyx Batryticatus	Jiangcan	15
Scorpion	Scorpio	Quanxie	6
Gambir Plant	Uncariae Ramulus cum Uncis	Gouteng	8
Resuscitative herbs			
Acorus Tatarinowii	Acori Tatarinowii Rhizoma	Shichangpu	16
Others			
Indian buead	Poria	Fuling	13
Salvia Root	Salviae Miltiorrhizae Radix Rhizoma	Danshen	7
Chinese Thorowax Root	Bupleuri Radix	Chaihu	6

### 3.3. Quality assessment of included studies

Thirteen studies^[13,14,16–20,22–24,26,27,29]^ utilizing the random number table method were assessed as low-risk. The other 6 studies^[11,12,15,21,25,28]^ only used the word “random” without a description of a specific method and were considered to be unclear.

One low-risk study^[13]^ mentioned the use of opaque envelopes for allocation concealment; whether allocation concealment was used was not reported in any of the remaining 18 papers. One of the study^[13]^ mentioned that the blind method could not be applied to researchers and subjects due to experimental reasons, which was rated as high risk. One study^[15]^ mentioned the use of a double-blind method, but did not provide a specific description. The remaining 17 papers did not mention the principle of blinding. Moreover, one study^[13]^ reported blinding of outcome assessors.

**Figure 2.**
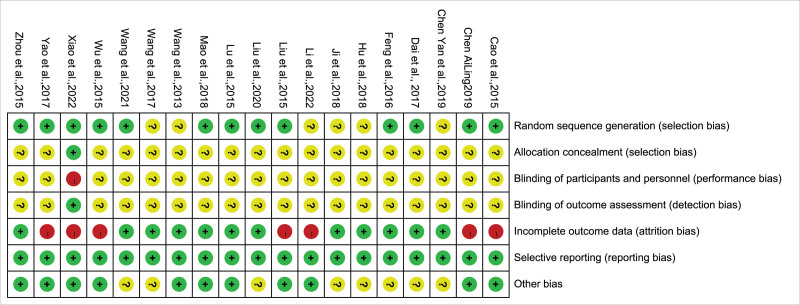
Summary chart of risk of bias assessment for included RCTs. RCTs = randomized controlled trials.

**Figure 3.**
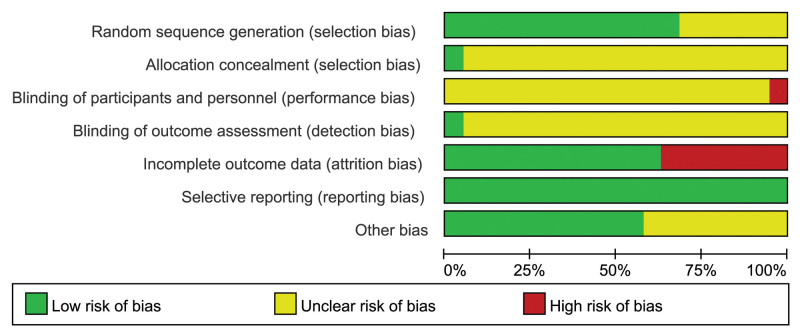
Percentage bar chart of risk of bias assessment for included RCTs. RCTs = randomized controlled trials.

Seven studies^[12–14,20,22,24]^ reported 44 participant withdrawals and documented their reasons. The remaining 18 studies did not report any participant withdrawal. All the 19 studies reported predefined outcome measures. Eight studies^[11,15,17,24–26,28,29]^ did not identify additional biases, the remaining 11 studies reported comparable baseline characteristics (Figs. 2 and 3).

## 4. Meta-analysis results

### 4.1. Clinical efficacy

**Figure 4.**
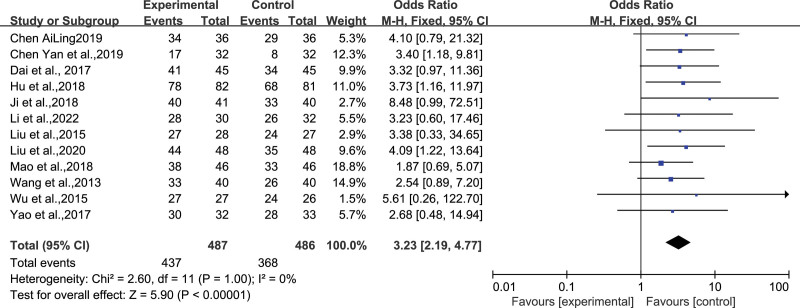
Forest plot depicting the clinical efficacy of WQPR therapy for epilepsy. WQPR=wind-quenching and phlegm-resolving.

Among all included studies, 12 RCTs (n = 487 in the experimental group and n = 486 in the control group) showed clinical efficacy.^[11,12,14,19–21,23–25,27–29]^ The heterogeneity analysis indicated homogeneity among the studies (*I*^2^ = 0%, *P* = 1.00) and warranted the fixed-effects model’s usage for amalgamation. The meta-analysis results demonstrated superior clinical efficacy in the experimental group’s treatment when compared to the control group, with a statistically significant difference(*OR* = 3.23, *95% CI* [2.19, 4.77], *Z* = 5.90, *P* < .00001) (Fig. 4).

### 4.2. Frequency of epileptic seizures

**Figure 5.**
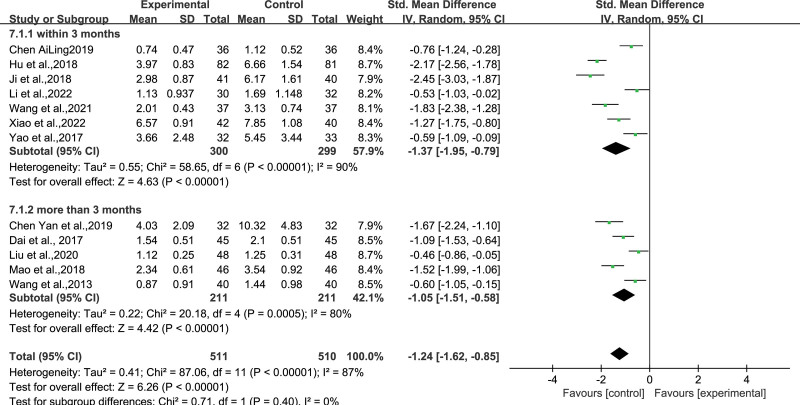
Forest plot depicting the frequency of epileptic seizures after WQPR therapy for epilepsy. WQPR=wind-quenching and phlegm-resolving.

Twelve studies^[11–14,20,21,24–29]^ that comprised 511 and 510 participants in the experimental and control groups utilized the frequency of epileptic seizures as an outcome measure. A random-effects model was utilized due to high inter-group heterogeneity (*I*^2^ = 95%, *P* < .00001).The pooled results revealed that WQPR therapy reduced the frequency of epileptic seizures compared with the control group (SMD = ‐1.24, *95% CI* [‐1.62, ‐0.85], *Z* = 6*.26*, *P* < .00001). A subgroup analysis was conducted according to the treatment duration; seven studies had interventions within 3 months and 5 had interventions for more than 3 months. The standardized mean differences in the frequency of epileptic seizures were significantly different between the WQPR therapy and the control groups within 3 months (SMD = ‐1.37, *95% CI* [‐1.95, −0.79], *Z* = 4.*63*, *P* < .00001). And more than 3 months [SMD = ‐1.05, *95% CI* (‐1.51, ‐0.58), *Z* = 4.42, *P* < .00001] of treatment, were consistent with the pooled results. After subgroup analysis based on the intervention time, the heterogeneity between the 2 groups remained high, suggesting that the intervention time may be one of the sources of heterogeneity. Meanwhile, the combined results of the 2 subgroups were statistically significant with SMD < 0, which was consistent with the total combined results, suggesting that treatment with WQPR therapy could reduce the frequency of seizures (Fig. 5).

### 4.3. Duration of seizures

**Figure 6.**
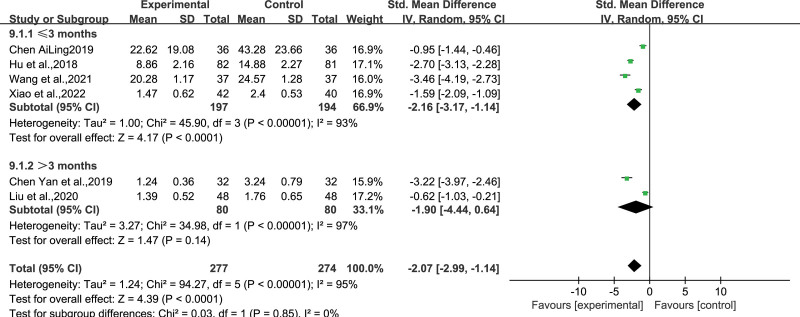
Forest plot displaying the duration of seizures after WQPR therapy for epilepsy. WQPR=wind-quenching and phlegm-resolving.

Six studies^[11,13,20,25,28,29]^ encompassing 277 and 274 participants in the experimental and control groups, respectively, reported the duration of epileptic seizures. Due to the high heterogeneity among the studies (*I*^2^ = 95%, *P* < .00001), a random-effects model was employed for analysis. Which revealed that WQPR therapy could reduce seizure duration compared to the control group (SMD = ‐2.07, *95% CI* [‐2.99, −1.14], *Z* = 4.39, *P* < .0001). A subgroup analysis was conducted according to the treatment duration that 4 studies had interventions within 3 months and 2 studies displayed interventions for >3 months. The standardized mean differences in the duration of seizures were significantly different between the WQPR therapy and control groups within 3 months (SMD = ‐2.16, *95% CI* [‐3.17, ‐1.14], *Z* = 4.17, *P* < .0001). At more than 3 months, no statistically significant difference was observed in the duration of epileptic seizures between the experimental and control groups (SMD = ‐1.90, *95% CI* [‐4.44, ‐0.64], *Z* = 1.47, *P* = .14). After subgroup analysis based on intervention time, the heterogeneity between the 2 groups remained high, suggesting that intervention time may be one of the sources of heterogeneity (Fig. 6).

### 4.4. QOLIE-31 scores

**Figure 7.**
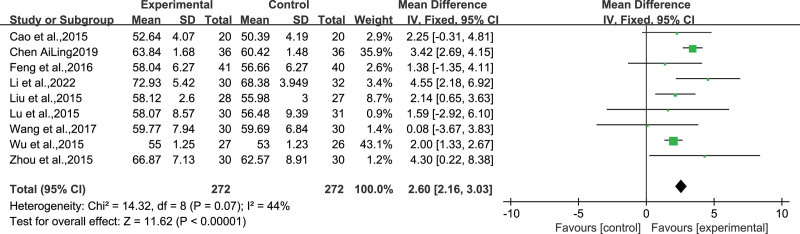
Forest plot for the meta-analysis of QOLIE-31 scores of WQPR therapy for epilepsy. QOLIE-31 scores=31-item Quality of Life Questionnaire in Epilepsy Inventory, WQPR=wind-quenching and phlegm-resolving.

Nine studies^[12,15–20,22,23]^ comprising 272 participants in both the experimental and control groups used the QOLIE-31 score as an outcome measure. A fixed-effects model was used for the analysis because of the mild heterogeneity observed among the studies(*I*^2^ = 43%, *P* = .08). The meta-analysis results demonstrated that the treatment group was more effective than the control group in improving the quality of life scores in patients with epilepsy(MD = 2.60, *95% CI* [2.16, 3.03], *Z* = *11.62*, *P* < .00001) (Fig. 7).

### 4.5. TCM symptom scores

**Figure 8.**
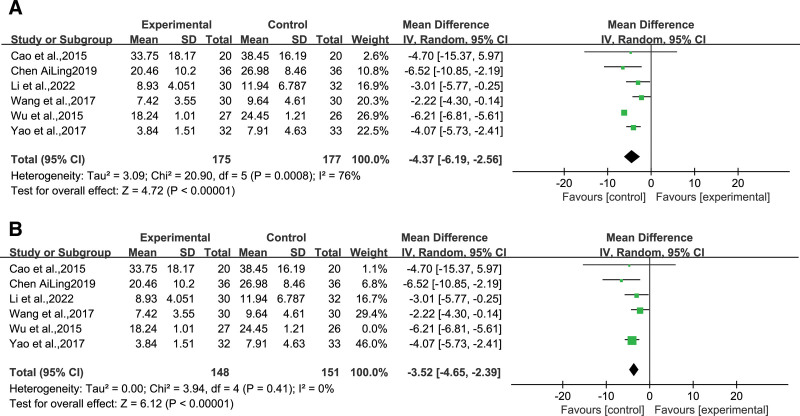
(A) Forest plot for the meta-analysis of TCM symptom scores after WQPR therapy for epilepsy. (B) Forest plot for the meta-analysis of after excluding the study by Wu. TCM = traditional Chinese medicine, WQPR = wind-quenching and phlegm-resolving.

Among all studies, 6^[12,14,15,20,22,23]^ employed the TCM symptom score as an outcome measure, comprising 175 and 177 participants in the experimental and control groups. Since significant heterogeneity was observed among the studies (*I*^2^ = 76%, *P* = .0008), a random-effects model was used for analysis. The results showed that, compared with the control group, the experimental group reduced the TCM symptom score more significantly [MD = ‐4.37, *95% CI* (‐6.19, ‐2.56), *Z* = 4.72, *P* < .00001] (Fig 8A). Sensitivity analysis revealed that after excluding the study by Wu et al (2015),^[23]^ the WQPR therapy group was more effective than the control group in lower TCM symptom scores[MD = ‐3.52, *95% CI* (‐4.65, ‐2.39), *Z* = *6.1*2, *P* < .00001] with no heterogeneity (*I*^2^ = *0*%) (Fig. 8B).

### 4.6. Adverse reactions

**Figure 9.**
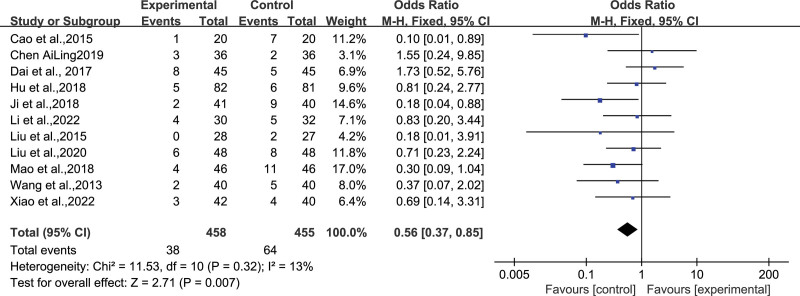
Forest plot displaying adverse reactions after TCM’s WQPR therapy for epilepsy. TCM = traditional Chinese medicine, WQPR = wind-quenching and phlegm-resolving.

Eleven studies^[12,13,19–22,24,25,27–29]^ reported the occurrence of adverse reactions, including 458 and 455 participants in the experimental and control groups. Owing to the low heterogeneity among the studies (*I*^2^ = 13%, *P* = .54), a fixed-effects model was used for result amalgamation. The meta-analysis results revealed a significant reduction in adverse reactions in the experimental group as compared with the control group (OR = 0.56, *95% CI* [0.37, 0.85], *Z* = 2.71, *P* = .007). The most common adverse reactions included dizziness, nausea, drowsiness, and gastrointestinal dysfunctions, like loose stools, abdominal distension, and slight loss of appetite. Since all the above adverse reactions were mild, they were relieved spontaneously or disappeared after symptomatic treatment. However, none of these studies reported serious adverse reactions (Fig. 9).

### 4.7. Publication bias

#### 4.7.1. Clinical efficacy

**Figure 10.**
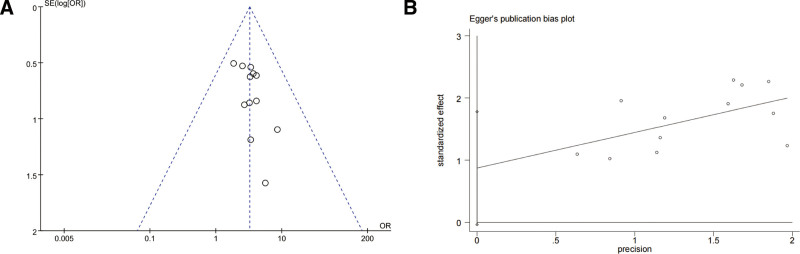
Funnel plot (A) and Egger funnel plot (B) for clinical efficacy.

Among the 12 studies, including clinical efficacy as the outcome measure, the funnel plot illustrated that the predominant distribution of effect estimates was within the confines of the 2 diagonal lines, and visual analysis indicated a slight publication bias, while no statistically significant publication bias was detected through Egger test (*P =* .058) (Fig. 10).

#### 4.7.2. Adverse reactions

**Figure 11.**
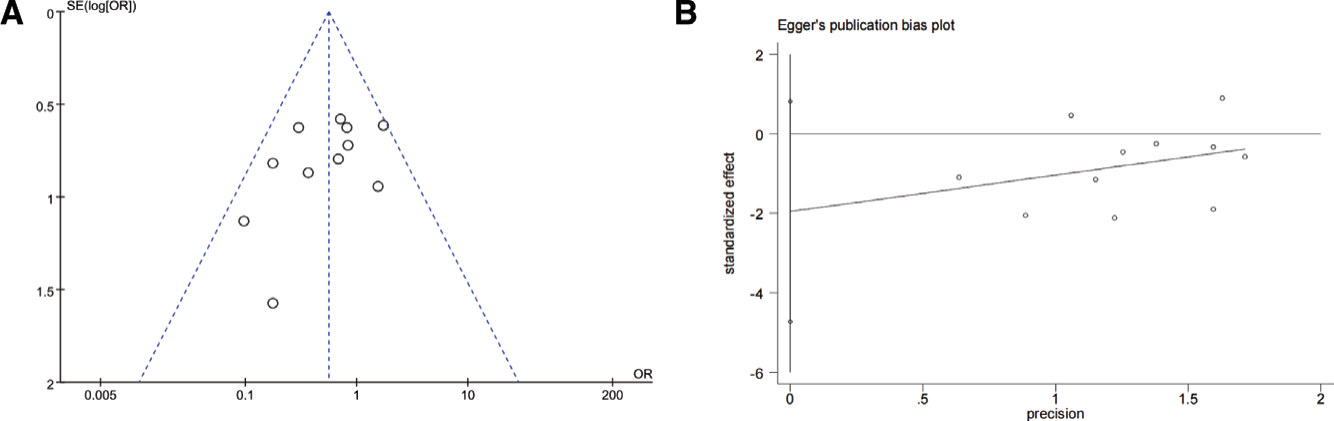
Funnel plot (A) and Egger funnel plot (B) for adverse reactions.

In the 11 studies reporting adverse reactions, the funnel plot showed that the distribution positions of the effect estimates were all within the 2 oblique lines. The majority of studies exhibited a bias toward the upper-middle region and displayed a roughly symmetrical distribution, the on left and right sides, suggesting that the possibility of publication bias was small. Meanwhile, and no statistically significant publication bias was detected through Egger test (*P* = .145) (Fig. 11).

### 4.8. Quality of evidence

**Figure 12.**
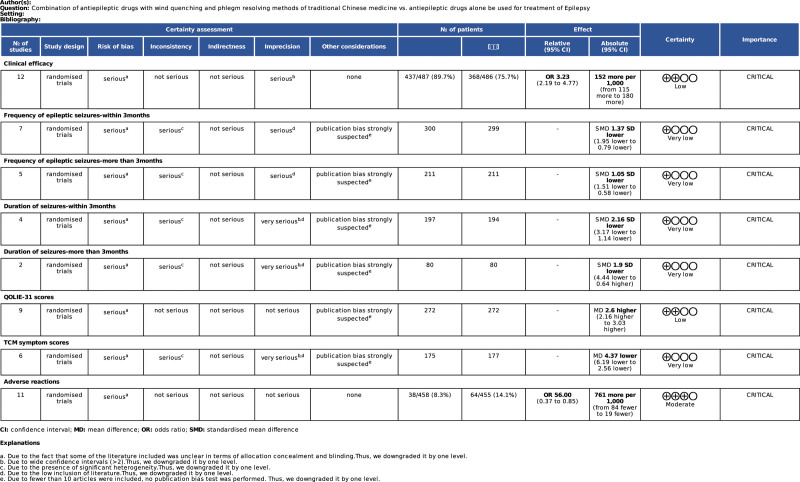
GRADE evidence profiles. GRADE = The Grading of Recommendations, Assessment, Development, and Evaluation system.

The GRADEpro system was used to assess the quality of evidence of the 8 outcomes. Adverse reactions were rated as moderate; clinical efficacy and QOLIE-31 scores were rated as low; frequency of epileptic seizures—within 3 months, frequency of epileptic seizures—>3 months, duration of seizures—within 3 months, duration of seizures—>3 months, and TCM symptom scores, were rated as very low. Due to the fact that some of the included literature was unclear in terms of allocation concealment and blinding, wide confidence intervals (>2), the presence of significant heterogeneity, and low inclusion, all contributed to lowering the GRADS level of evidence (Fig. 12).

## 5. Discussion

Diverse manifestations of epileptic seizures include sensory, motor, mental, behavioral, and autonomic dysfunctions. Frequently accompanied by comorbidities like anxiety, depression, and sleep disorders, epilepsy significantly impairs a patient’s quality of life. With the biomedical paradigm making way for the biopsychosocial medical model, the contemporary treatment for epilepsy aims to enhance overall patient quality of life and seizure management.

According to TCM, epilepsy is localized in the brain and closely linked to the heart, liver, spleen, and kidneys. The pathogenesis involves the loss of control over vital mechanisms, leading to disease onset. Subsequent investigators have often associated epilepsy with pathogenic factors like wind and phlegm. As documented in the literature, treatment primarily involves medications based on the WQPR approach. Commonly used herbs include Tall Gaxtraodia Tuber, Bile Arisaema, Acorus Tatarinowii, Pinellia Tuber, Indian buead, Stiff Silkworm, Scorpion, Tangerine Peel, and Gambir Plant. In clinical practice, the application of these herbs is adjusted based on specific changes in the patient’s condition.

With the advancement of modern pharmacology, the components of traditional Chinese medicine are been thoroughly investigated. In Acorus Tatarinowii, the most abundant active ingredients are α-asarone and β-asarone. A study by Lai Xin^[30]^ suggested that α-asarone can attenuate post-seizure conditions in clinical settings by activating of brain tissue nuclear factor-kappa B (NFκB) and significantly reducting inducible nitric oxide synthase (iNOS) and cyclooxygenase-2 (COX-2) expression. By inhibiting the activation of hippocampal CA1, CA3, and adjacent cortical glial cells, it alleviates neuroinflammation. Similarly, β-asarone significantly reduced the protein expressions of Caspase-1 and IL-18, inhibited pyroptosis, reduced cell damage, and exerted antioxidant effects. It also lowers cell supernatant’s lactate dehydrogenase and glutathione activities, and enhances the activities of hydrogen peroxide, thereby exhibiting antioxidant properties.^[31]^

Gastrodin and Parishin B are the primary active components of the Tall Gaxtraodia Tuber. Zheng et al^[32]^ revealed that gastrodin prolonged the latency of epileptic waves, reduced the frequency of major seizures, increased the number of surviving neurons, decreased apoptotic neurons, and exerted antiepileptic effects in rats. It can also inhibit the activation of astrocytes, thereby alleviating epileptic seizures. Wang et al^[33]^ explored the effects of the active component Parishin B in Rhizoma Gastrodiae on epileptic behaviors in mice. Their results suggested that Parishin B displayed potential antiepileptic activity because of its interaction with targets such as NRAS, HRAS, FGFR3, regulation of PI3K-Akt, Rap1, VEGF neuroinflammation reduction, and modulation of the neuronal autophagy signaling pathway, thereby exerting an antiepileptic effect.

Lei et al^[34]^ discovered that Gambir Plant, a major bioactive component in Radix Ophiopogonis, reduced hippocampal tissue damage in epileptic rats, decreased the frequency of epileptic seizures, significantly shortened the seizure duration, increased EEG frequency, reduced wave amplitude, enhanced cognitive and learning abilities, and decreased epileptic seizures.

Additionally, insect-based medicines with innate wind-dispelling and convulsion-alleviating effects, possess blood-activating and stasis-dissolving properties.^[35]^ Bombyx Batryticatus, also known as Jiangcan, exhibits anticonvulsive, sedative-hypnotic, antibacterial, anticoagulant, hypoglycemic, and lipid-lowering effects. Thus, injecting *Bombyx batryticatus* extract into animal models significantly reduced the thrombus weight. Scorpion-based toxins extracted from scorpion demonstrates strong antiepileptic properties, significantly reduce the excitability and sensitivity of hippocampal neurons to epileptic seizures. Moreover, by inhibiting hippocampal astrocyte proliferation and hypertrophy, it alleviates damage to the hippocampal neurons.^[36]^

We systematically evaluated the efficacy and safety of TCM using the WQPR approach for epilepsy treatment. Given the diverse intervention periods in the literature, which may have influenced the results, we conducted subgroup analyses based on the intervention time. Our meta-analysis results showed that, compared to AEDs, the WQPR approach can significantly improve clinical efficacy, reduce seizure frequency, shorten seizure duration, improve patients’ quality of life, and alleviate TCM symptoms. Furthermore, the most common adverse reactions include dizziness, nausea, drowsiness, and gastrointestinal dysfunctions, like loose stools, abdominal distension, and slight loss of appetite. As none of the studies reported serious adverse reactions, all mild adverse reactions disappeared with or without medical interventions; therefore, the WQPR approach therapy appears to be a safe approach for treating epilepsy.

In this study, the GRADEpro system was used to assess the quality of evidence for the 8 outcomes. The results showed that adverse reactions were rated as moderate; clinical efficacy, QOLIE-31 scores were rated as low; frequency of epileptic seizures—within 3 months, frequency of epileptic seizures—>3 months, duration of seizures—within 3 months,duration of seizures—>3months,as well as TCM symptom scores, were rated as very low. Due to the fact that some of the included literature was unclear in terms of allocation concealment and blinding, wide confidence intervals (>2), the presence of significant heterogeneity, and low inclusion, all contribute to lowering the GRADS level of evidence. Thus, further high-quality, well-designed RCTs with long-term follow-up are still required.

Nevertheless, this study has several limitations. First, due to the lack of relevant literature reported abroad, the included studies were all conducted in China, which may have publication bias and need to be further supplemented. Second, some studies lacked detailed descriptions of random sequence generation and did not specify the implementation of blinding and allocation concealment, thus potentially impacting the results. Third, variations in the composition, dosage, formulation, and decoction time of the included herbal remedies may have introduced bias into the results. Thus, further high-quality, well-designed RCTs with long-term follow-up are still required to further validate the accuracy of the conclusions on the efficacy and safety of traditional Chinese medicine for the treatment of epilepsy by WQPR.

## 6. Conclusion

In summary, our results indicate that TCM utilizing the WQPR approach can be advantageous for treating epilepsy. The clinical efficacy and safety of the treatment group were superior to the those of control group treated solely with AEDs. Additionally, the WQPR approach is safe for clinical applications. However, the majority of the included clinical trials exhibited suboptimal methodological quality and might have impacted the our reliability of our results. Therefore, future efforts should include large-sample, multicenter, double-blind RCTs to objectively evaluate the clinical efficacy of TCM’s WQPR approach in the treatment of epilepsy.

## Acknowledgments

We are grateful for the support from the National Natural Science Foundation of China (82060863), Science and Technology Projects of Guizhou Province (Qiankeheji-zk [2021] General 500), Research Project of the Second Affiliated Hospital of Guizhou University of TCM GZEYK [2020]11, and Research Project of Guizhou University of Traditional Chinese Medicine [2019]20.

## Author contributions

**Conceptualization:** Yufen Cai, Xiaofang He, Hui Yang, Lin Zhang.

**Data curation:** Xiaofang He, Yibo Liu, Yanju Zhang.

**Formal analysis:** Yufen Cai, Liting Ao, Yibo Liu.

**Funding acquisition:** Lin Zhang.

**Investigation:** Xiaofang He, Liting Ao, Yanju Zhang.

**Methodology:** Yufen Cai, Xiaofang He, Liting Ao.

**Project administration:** Yufen Cai, Hui Yang, Lin Zhang.

**Resources:** Xiaofang He, Liting Ao, Lin Zhang.

**Software:** Yufen Cai, Xiaofang He, Lin Zhang.

**Supervision:** Hui Yang, Lin Zhang.

**Validation:** Yufen Cai, Yanju Zhang, Hui Yang.

**Visualization:** Yufen Cai, Yibo Liu, Lin Zhang.

**Writing – original draft:** Yufen Cai, Xiaofang He, Liting Ao, Yibo Liu, Yanju Zhang.

**Writing – review & editing:** Hui Yang, Lin Zhang.
